# Supplementary material for: HIVprotI: an integrated web based platform for prediction and design of HIV proteins inhibitors
Source: J Cheminform. 2018 Mar 9;10:12. doi: 10.1186/s13321-018-0266-y (PMC5845081; doi:10.1186/s13321-018-0266-y)
Supplement: Supplementary file 2 — Additional file 2. Source code of HIVProtI web server. [file 13321_2018_266_MOESM2_ESM.zip › HIVprotI_Source-code/hivprotI/weka-3-6-6/doc/weka/core/Debug.Timestamp.html]

Debug.Timestamp


---


|  |  |  |
| --- | --- | --- |
| **Overview** \| **Package** \| **Class** \| **Tree** \| **Deprecated** \| **Index** \| **Help** \| **Weka's home** | |  |
| **PREV CLASS**   **NEXT CLASS** | **FRAMES**    **NO FRAMES**     **All Classes** |  |
| SUMMARY: NESTED \| FIELD \| CONSTR \| METHOD | DETAIL: FIELD \| CONSTR \| METHOD |  |


---


## weka.core Class Debug.Timestamp

```
java.lang.Object
  weka.core.Debug.Timestamp
```

**All Implemented Interfaces:**: java.io.Serializable, RevisionHandler

**Enclosing class:**: Debug

---

``` public static class Debug.Timestamp extends java.lang.Object implements java.io.Serializable, RevisionHandler ```

A class that can be used for timestamps in files, The toString() method
simply returns the associated Date object in a timestamp format. For
formatting options, see java.text.SimpleDateFormat.

**Version:**
:   $Revision: 7519 $

**Author:**
:   FracPete (fracpete at waikato dot ac dot nz)

**See Also:**: `SimpleDateFormat`, Serialized Form

---

| **Field Summary** | |
| --- | --- |
| `static java.lang.String` | `DEFAULT_FORMAT`             the default format |


**Constructor Summary**

`Debug.Timestamp()`
  
          creates a timestamp with the current date and time and the default
format.

`Debug.Timestamp(java.util.Date stamp)`
  
          creates a timestamp with the given date and the default format.

`Debug.Timestamp(java.util.Date stamp,
java.lang.String format)`
  
          creates a timestamp with the given date and format.

`Debug.Timestamp(java.lang.String format)`
  
          creates a timestamp with the current date and time and the specified
format.


| **Method Summary** | |
| --- | --- |
| `java.lang.String` | `getFormat()`             returns the current timestamp format |
| `java.lang.String` | `getRevision()`             Returns the revision string. |
| `java.util.Date` | `getStamp()`             returns the associated date/time |
| `void` | `setFormat(java.lang.String value)`             sets the format for the timestamp |
| `java.lang.String` | `toString()`             returns the timestamp as string in the specified format |

**Methods inherited from class java.lang.Object**

`equals, getClass, hashCode, notify, notifyAll, wait, wait, wait`

**Field Detail**

### DEFAULT\_FORMAT

```
public static final java.lang.String DEFAULT_FORMAT
```

:   the default format

    **See Also:**: Constant Field Values


**Constructor Detail**

### Debug.Timestamp

```
public Debug.Timestamp()
```

:   creates a timestamp with the current date and time and the default
    format.

---


### Debug.Timestamp

```
public Debug.Timestamp(java.lang.String format)
```

:   creates a timestamp with the current date and time and the specified
    format.

    **Parameters:**: `format` - the format of the timestamp **See Also:**: `SimpleDateFormat`

---


### Debug.Timestamp

```
public Debug.Timestamp(java.util.Date stamp)
```

:   creates a timestamp with the given date and the default format.

    **Parameters:**: `stamp` - the associated date/time for the timestamp

---


### Debug.Timestamp

```
public Debug.Timestamp(java.util.Date stamp,
                       java.lang.String format)
```

:   creates a timestamp with the given date and format.

    **Parameters:**: `stamp` - the associated date/time for the timestamp: `format` - the format of the timestamp **See Also:**: `SimpleDateFormat`


**Method Detail**

### setFormat

```
public void setFormat(java.lang.String value)
```

:   sets the format for the timestamp

    :   **Parameters:**: `value` - the format string **See Also:**: `SimpleDateFormat`

---


### getFormat

```
public java.lang.String getFormat()
```

:   returns the current timestamp format

    :   **Returns:**: the current format

---


### getStamp

```
public java.util.Date getStamp()
```

:   returns the associated date/time

    :   **Returns:**: the timestamp value

---


### toString

```
public java.lang.String toString()
```

:   returns the timestamp as string in the specified format

    :   **Overrides:**: `toString` in class `java.lang.Object`
    :   **Returns:**: the timestamp as string

---


### getRevision

```
public java.lang.String getRevision()
```

:   Returns the revision string.

    :   **Specified by:**: `getRevision` in interface `RevisionHandler`
    :   **Returns:**: the revision


---


|  |  |  |
| --- | --- | --- |
| **Overview** \| **Package** \| **Class** \| **Tree** \| **Deprecated** \| **Index** \| **Help** \| **Weka's home** | |  |
| **PREV CLASS**   **NEXT CLASS** | **FRAMES**    **NO FRAMES**     **All Classes** |  |
| SUMMARY: NESTED \| FIELD \| CONSTR \| METHOD | DETAIL: FIELD \| CONSTR \| METHOD |  |


---
